# Population Dynamics and the Microbiome in a Wild Boreal Mammal: The Snowshoe Hare Cycle and Impacts of Diet, Season and Predation Risk

**DOI:** 10.1111/mec.17629

**Published:** 2024-12-19

**Authors:** Mason R. Stothart, Sophia Lavergne, Laura McCaw, Hardeep Singh, Wilfred de Vega, Katherine Amato, Jocelyn Poissant, Rudy Boonstra

**Affiliations:** ^1^ Faculty of Veterinary Medicine University of Calgary Calgary Alberta Canada; ^2^ Department of Biology University of Oxford Oxford UK; ^3^ Department of Biological Sciences University of Toronto Scarborough Toronto Ontario Canada; ^4^ Department of Anthropology Northwestern University Evanston Illinois USA

**Keywords:** 16S rRNA, food limitation, gut, health, HPA axis, stress, wildlife

## Abstract

The North American boreal forest is a massive ecosystem, and its keystone herbivore is the snowshoe hare ( *Lepus americanus* ). Hares are exposed to considerable environmental extremes in diet and weather, food availability, and predation risk. Gut microbiomes have been suggested to facilitate adaptive animal responses to environmental change, but severe environmental challenges to homeostasis can also disrupt host‐microbiome relationships. To better understand gut microbiome contributions to animal acclimation, we studied the faecal bacterial microbiome of wild hares across two types of extreme environmental change that are integral to their natural history: (1) seasonal transitions between summer and winter, and (2) changes over the ~10 year ‘boom‐bust’ population cycles that are characterised by shifting food resource availability and predation pressure. When compared to summer, hares in winter had lower bacterial richness and were depleted in 20 families (including Oxalobacteraceae and Christensenellaceae) but enriched for Ruminococcaceae (a family which contains plant fibre degrading bacteria) alongside nine other bacterial groups. Marked bacterial microbiome differences also occurred across phases of the population cycle. Bacterial microbiomes were lower in richness and compositionally distinct in the peak compared to the increase or decline phases of the population cycle. Direct measures of host physiology and diet quality (faecal fibre contents) most strongly supported food resource availability as a mechanism underlying phase‐based differences in bacterial communities, but faecal fibre contents could not fully account for bacterial microbiome variation across phases.

## 1 Introduction

Wild animals have evolved an array of physical, functional and behavioural traits to maximise fitness in challenging environments, allowing them to cope with conspecifics, predators, seasonal fluctuations and changing resource availability. Increasingly, the composition and function of an organism's microbiome—and the gut microbiome in particular—is being recognised as a key determinant of individual fitness, population health and adaptive phenotypic patterns (Bordenstein and Theis 2015; Moloney et al. 2014; Risely et al. 2023; Stothart et al. 2024). Through their metabolic activities, the microbial communities within mammalian intestinal tracts play critical roles in regulating host development, immunity, metabolism and behaviour (Kohl 2018; McFall‐Ngai et al. 2013). The microbiome also performs numerous ecological functions (McKenney et al. 2018). These include widening the dietary niches and metabolic potential of animals that could not otherwise exploit certain resources due to, for example, high levels of indigestible chitin, cellulose and lignin (Barboza, Parker, and Hume 2009; Chivers and Langer 1994; Moeller and Sanders 2020; Teullet et al. 2023), or the presence of toxic plant secondary metabolites (Blyton et al. 2019; Kohl et al. 2014; McArthur et al. 2014).

The microbiome can be viewed as a phenotypic extension of the host, which is shaped by both host and environmental factors (Kolodny and Schulenburg 2020). Coupled with its ability to influence host phenotype, the microbiome's dynamic nature suggests that it could be a critical enabler of rapid organismal acclimation to environmental change (Alberdi et al. 2016) that is either novel (Stothart and Newman 2021) or recurrent over circadian (Risely et al. 2021) and seasonal (Björk et al. 2022; Regan et al. 2022; Stothart et al. 2023) timescales. However, changes in the microbiome that co‐occur with environmental changes may not necessarily be adaptive. For example, stressor‐related disruption of host homeostasis can push microbiomes into a disease state (Zaneveld, McMinds, and Vega Thurber 2017). Here we investigated the gut microbiome dynamics of a wild population of snowshoe hares ( *Lepus americanus* ) in southwestern Yukon (Canada) that experiences annual cycles of seasonal change, in addition to a ~10 year ‘boom‐bust’ population cycle that are consequential for hare health as well as food web dynamics of the broader ecosystem (Krebs, Boonstra, and Boutin 2018).

The snowshoe hare is a keystone herbivore in the boreal forests of North America (> 5,000,000 km^2^) and its existence is characterised by extreme variation in its physical and social environments. This northern region experiences major seasonal fluctuations in precipitation, daylight, and temperature, that in turn determine seasonal plant availability and hare diet (Krebs, Boonstra et al. 2001; Krebs, Dale et al. 2001). In winter, hares subsist primarily on high‐fibre woody browse from willow, birch and spruce species (Bookhout 1965; Hodges and Sinclair 2003; Sinclair and Smith 1984). The transition to spring is associated with a 5‐fold increase in herbaceous foods and a decline in the consumption of fibrous bark and twigs (from 43% to 28% of the diet; Smith, Hubartt, and Shoemaker 1980), culminating in a more varied summer diet enriched with leaves, forbs and grasses (Seccombe‐Hett and Turkington 2008). Concurrent with changes in the availability of food species across the annual cycle, the snowshoe hare digestive system is seasonally remodelled, with pancreas mass increasing and cecal length decreasing from winter to spring (Smith, Hubartt, and Shoemaker 1980). Together, seasonal patterns in snowshoe hare diet and physiology are likely to affect the gut microbiome, and seasonal remodelling of the gut microbiome might be necessary for hares to meet nutritional demands in a shifting dietary landscape.

The most prominent feature of the snowshoe hare's biology is its 8–11 year ‘boom‐bust’ cycle in population abundance. Hare numbers fluctuate up to 40‐fold across the cycle and are closely tracked (with a 1‐ to 2‐year lag) by those of their specialist predator the Canada lynx ( *Lynx canadensis* ; Krebs et al. 1995; Krebs, Boonstra, and Boutin 2018). Predators regulate hare populations via direct mortality as well as through sublethal effects on their physiology and reproduction (Boonstra et al. 1998; Sheriff, Krebs, and Boonstra 2010). A hare's risk of being predated is greatest during the decline phase, when lynx density and the ratio of predators‐to‐prey are at their maximum (Krebs et al. 1995). Though large‐scale experimentation has conclusively found food‐related hypotheses alone are insufficient explanations for the hare cycle (Krebs, Boutin, and Gilbert 1986), investigations have nonetheless demonstrated that hares experience variable food resources across the cycle, as supported by evidence for both absolute food restrictions due to over‐browsing (Krebs, Boonstra et al. 2001; Krebs, Dale et al. 2001; Sinclair, Krebs, and Smith 1982; Sinclair et al. 1988; Smith et al. 1988) and increased starvation‐related deaths at peak hare densities (Boutin et al. 1986; Hodges, Boonstra, and Krebs 2006). Fluctuations in food availability across the population cycle are accompanied by changes in anti‐nutritional plant secondary metabolites (PSMs; tannins, terpenes and phenols) contained within core food items, as plant shoots of differing developmental maturity are chemically defended to differing extents (Deangelis et al. 2015; Sinclair et al. 1988). Microbiota in the snowshoe hare gut could play an important functional role by facilitating hare subsistence on plants laden with anti‐nutritious compounds (Blyton et al. 2019; Kohl et al. 2014).

Previous investigations have also demonstrated that heightened predation risk during the decline phase of the cycle can lead to chronic stress effects and dampened reproductive output in hares (Boonstra et al. 1998; Sheriff, Krebs, and Boonstra 2009, 2010)—effects that appear proportional to the degree of risk (MacLeod et al. 2018) and endure even after the actual risk of predation has abated (Sinclair et al. 2003). Variable predator density across the cycle can also influence hare nutrition as individuals adjust their foraging behaviour under heightened risk in favour of safer locations with less nutritive or palatable resources (Hik 1995). This predator‐sensitive foraging hypothesis has found recent support in studies that have shown that wild hares supplemented with food spend less time foraging per day (Majchrzak et al. 2022), and that hares reduce foraging time for up to 10 h after encountering a lynx (Shiratsuru et al. 2021).

Mounting evidence supports the existence of a relationship between hypothalamic–pituitary adrenal (HPA) axis activity and the microbiome (Petrullo et al. 2022; Stothart, Palme, and Newman 2019; Sudo et al. 2004), and as herbivores, hares are obligately reliant on gut microbiota to extract nutrients from their plant‐based diet. The chronic stress effects that derive from perceived predation risk could secondarily influence hare nutrition if it disrupts the gut microbiome. Such disruptions could decrease digestive efficiency, increase the risk of opportunistic pathogen emergence in the gut (Spragge et al. 2023), or leave hares vulnerable to plant toxins (Kohl et al. 2014; Moloney et al. 2014), and thereby contribute to increased risk of starvation and predation that occurs during the peak and decline phases of the hare cycle (Boutin et al. 1986; Hodges, Boonstra, and Krebs 2006). Previous observations that chronic stress depresses hare condition and reproduction during the peak and decline phases of the cycle (Boonstra et al. 1998; Sheriff, Krebs, and Boonstra 2009, 2010), may therefore be partly linked to the microbiome. Given regular and marked fluctuations in their environment, snowshoe hares represent an excellent species in which to investigate plasticity of the gut microbiome and connections of the gut microbiome to host stress physiology.

We conducted a 6‐year investigation of wild snowshoe hares to characterise seasonal and cyclical patterns of variation in their gut bacterial microbiome. In our seasonal comparison (Study 1), we contrasted the summer and winter bacterial microbiome of hares across 3 years of population increase. The relative risk of predation is low throughout the increase phase, but the nutritional quality and diversity of hare diets are predictably higher in summer than winter (Seccombe‐Hett and Turkington 2008; Wolff 1978). Within our seasonal comparison, we therefore predicted higher microbial diversity in summer‐collected samples, but that samples collected in winter would be functionally enriched for fibre and lignocellulose degrading microbes as hares become reliant on high‐fibre woody browse (Bookhout 1965; Hodges and Sinclair 2003; Smith et al. 1988).

In our cyclical comparison (Study 2), we assessed variation in gut microbiota over 6 winters that spanned the increase, peak and decline phases of the hare population cycle. We also assessed the ability of more proximate measured aspects of host condition (haematocrit), immunity (neutrophil: lymphocyte [N:L] ratio) and HPA axis function (faecal cortisol metabolite concentration [FCM], blood glucose concentrations during dexamethasone‐adrenocorticotropic hormone challenge; Lavergne et al. 2021)—as well as an index of dietary quality, faecal fibre content (Hodges and Sinclair 2003)—to explain observed patterns of microbial diversity across the hare population cycle. For our cyclical analysis, we predicted that if phase‐based variation in the bacterial microbiome was the result of perceived predation risk (and its associated impacts on hare stress physiology), then a successional shift in microbiota community structure, or increases in beta diversity dispersion (Zaneveld, McMinds, and Vega Thurber 2017), would be observed from the increase to the decline phases of the population cycle, as predator densities increase relative to hare population size (Boonstra et al. 1998; Krebs, Boonstra et al. 2001). If cyclical differences in the bacterial microbiome are instead primarily attributable to diet, then we predicted that the bacterial microbiome would differ between all three phases. First, we predicted greater representation of plant fibre degrading gut microbes in the peak than increase or decline phases, since snowshoe hares consume less digestible foods during the peak phase (young white spruce [ *Picea glauca* ] and large diameter deciduous stems), after depleting preferred winter resources (small stems of dwarf birch [ *Betula glandulosa* ] and grey‐leaved willow [ *Salix glauca* ]) at peak hare densities (Bryant et al. 1994; Hodges and Sinclair 2003; Krebs, Boonstra et al. 2001; Krebs, Dale et al. 2001; Sinclair et al. 1988; Smith et al. 1988). Second, we predicted that microbes capable of degrading plant secondary metabolites (PSMs) would be more abundant during the decline phase than the increase or peak phases of the population cycle, since defensive chemicals are found at higher concentrations within the young stems rapidly produced by trees following peak hare browsing and subsequent population decline (Bryant et al. 1985; Deangelis et al. 2015; Smith et al. 1988). We directly tested the non‐mutually exclusive hypotheses that chronic stress and/or diet contributed to phase‐based variation in the gut bacterial microbiome, by using direct measures of diet quality and host physiology across the snowshoe hare population cycle.

## 2 Materials and Methods

### 2.1 Study Population and Sample Collection

**FIGURE 1:**
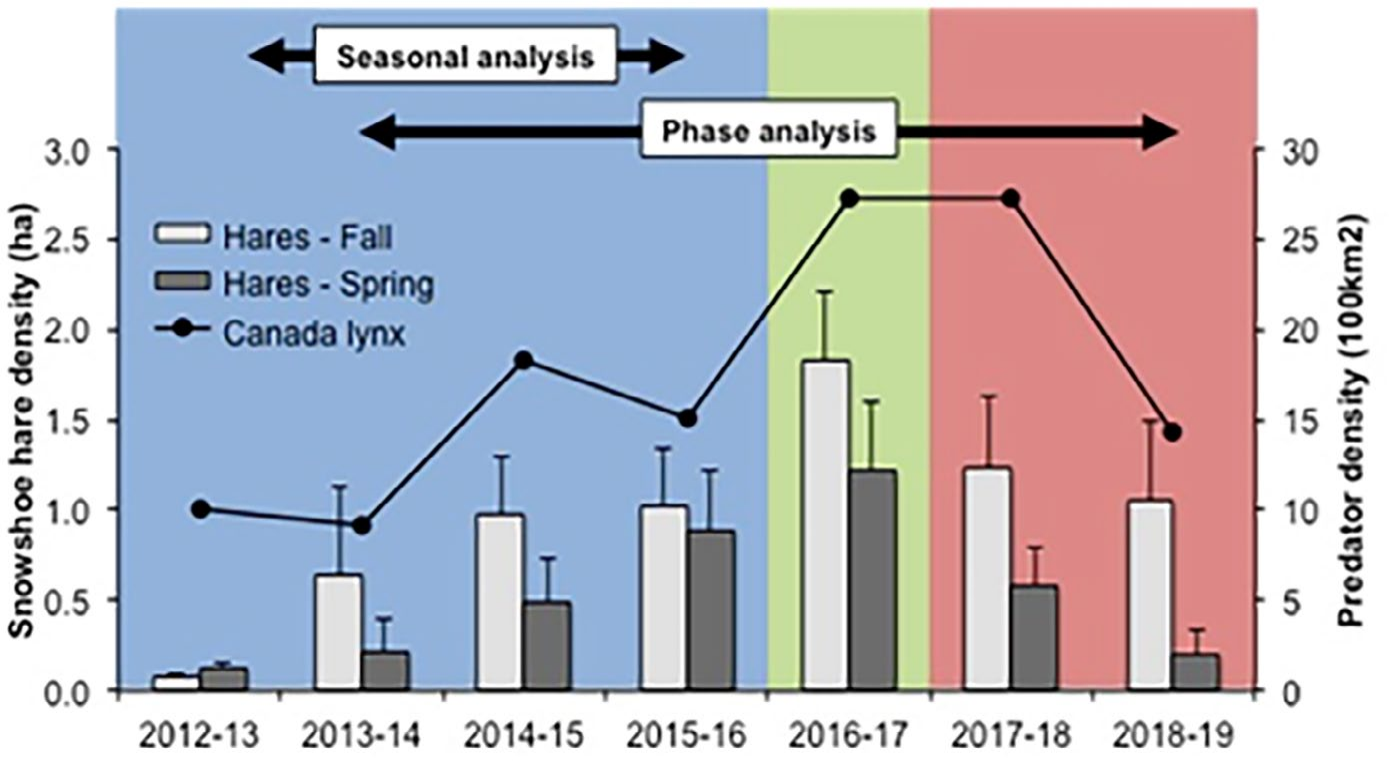
Density estimates of snowshoe hares in the fall and following spring, and Canada lynx in the intervening winter at Kluane during the study period. Shading delineates the increase (blue), peak (green) and decline (red) phases of the hare cycle.

We studied the gut bacterial microbiomes of adult snowshoe hares from the Shakwak Trench east of Kluane Lake in the southwestern Yukon (61° N, 138° W). The population inhabits the boreal forest along a 30‐km stretch of the Alaska Highway, between the Arctic Institute of North America's Kluane Lake Research Station and the Jarvis River (Figure S1). Hares in this region have been continuously monitored since 1977 (over 45 years) (Krebs, Boonstra et al. 2001; Krebs, Boonstra, and Boutin 2018). Density estimates for both hares and their mammalian predators over the course of our study period were provided courtesy of the Kluane Community Ecological Monitoring Project (CEMP; Figure 1; Krebs et al. 2022). The finite rate of annual change in spring hare density was used to distinguish the phases of the cycle (Keith 1990). Snowshoe hare population densities were estimated twice annually in September/October and March/April using capture‐mark‐recapture population surveys across two 36‐ha monitoring grids (Silver and Sulphur grids) containing 172 livetraps (Figure S1). Live trapping and tagging occurred across 2–3 trapping nights per session (Hodges et al. 2001). The maximum likelihood spatial estimator from DENSITY 4.4 was used to estimate hare population density during each trapping session (Efford, Borchers, and Byrom 2009). Lynx abundance was estimated each winter using tracks observed along a 22 km transect within 24–48 h following fresh snowfall between October and April of the focal year (Lavergne et al. 2021).

We conducted overnight hare trapping sessions using Tomahawk livetraps (No. 106, Tomahawk, WI, USA) baited with rabbit chow (Unifeed, Okotoks, AB, Canada) and a slice of apple in summer or snow in winter for hydration. Traps were set along paths, old roads and cutlines along either side of the Alaska Highway, with consistent trapping locations used across all study years. For all individuals captured, the time in trap was < 8 h. Summer sampling took place in early July (2013–2015). Hares were measured for body mass and right hind foot length (index of skeletal size), assessed for sex and reproductive status, given an identifying ear tag, and then released. Winter sampling took place in late February (2014–2019), with hares assessed as above and then transported to the Kluane Lake Research Station for in‐depth analyses of hypothalamic‐pituitary‐adrenal (HPA) axis function.

Hard faecal pellets (not cecotropes) were collected from below each trap (cleared forest floor in summer, snowpack in winter) at the time of hare processing. All faecal samples were at ambient temperatures from the time of defecation until early morning trap checking sessions, then transferred to a −20**°**C freezer for storage. Frozen samples were transported to the University of Toronto Scarborough where they were lyophilized for 14–18 h (FreeZone 4.5L, LabConco, MO, USA), manually homogenised in liquid nitrogen using a mortar and pestle, then stored at −20**°**C prior to FCM and fibre content analysis, as well as bacterial microbiome characterisation (Bensch et al. 2022; Gavriliuc et al. 2021).

### 2.2 Physiological Measures

For greater detail on how physiological measures were collected, and their interpretation, see Lavergne et al. (2021). In brief, haematocrit (percent of total blood volume attributable to red blood cells) estimates were obtained from the means of duplicate blood samples collected in 75 μL capillary tubes and centrifuged at 13,460 g for 8 min. Haematocrit is commonly used as haematological indicator of animal condition (Johnstone, Lill, and Reina 2012).

Duplicate blood smears were likewise collected and stained with Diff‐Quick (Dade International Inc., Florida, USA) to differentiate white blood cell types and obtain measurements of neutrophil: lymphocyte ratios within subsets of 100 leukocytes. The ratio of neutrophil to lymphocytes provides an immunological indicator of chronic stress and infection (Johnstone, Lill, and Reina 2012).

Faecal cortisol metabolites were extracted from 60 mg of frozen faeces by incubating samples at room temperature on an orbital shaker (30 min at 1500 rpm) in 1 mL of 80% methanol. Samples were then centrifuged for 15 min at 2500 g and 100 μL of the extraction supernatant was diluted [1:10] in assay buffer. An 11‐oxoaetiocholanolone enzyme immunoassay validated for snowshoe hares was used to quantify FCM concentration in faeces (Sheriff et al. 2009). FCM concentration provides an integrated measure of HPA axis activity in the days preceding sample collection. Notably, these measures do not reflect trapping stress, since it takes 8–10 h for increases in circulating cortisol to be reflected in snowshoe hare FCM estimates (Sheriff et al. 2009).

Circulating blood glucose concentrations (area under the curve) during dexamethasone‐adrenocorticotropic hormone challenges were quantified using a FreeStyle glucometer. This physiological measure reflects the capacity of hares to mobilise glucose during a controlled hormone challenge (Boonstra et al. 1998).

### 2.3 Faecal Fibre Analysis

An acid‐pepsin digestion protocol was used to quantify the fibre content of winter‐collected faecal samples as an index of hare nutrition. Only winter collected samples were assayed due to limited resources, sample material, and to allow for comparisons with physiological measures; however, faecal fibre contents are known to be greater in the winter when compared to summer (Hodges and Sinclair 2003). To estimate the proportion of dietary fibre consumed by hares, duplicate samples of 100 mg of lyophilized ground faeces from every sample was digested in 10 mL of a pepsin and hydrochloric acid solution at 37°C for 48 h, filtered through pre‐weighed Whatman #2 qualitative filter paper (Whatman, CAT#1002‐042) under vacuum, and then the remaining particulate matter was dried in a 100°C oven for 48 h and reweighed (Larter 1992). The fibre index was then calculated as (amount remaining/starting amount) × 100% (Seccombe‐Hett and Turkington 2008). The acid‐pepsin digestion protocol we used can return different estimates of fibre content for *Salix* spp. forage (Larter 1992) when compared to other methods (e.g., acid detergent fibre procedures), but in vivo validations of this acid‐pepsin digestion protocol among snowshoe hare fed natural forage species (including *Salix glauca* ) show a strong positive correlation between faecal fibre content and consumed dietary fibre (*R* ^2^ = 0.82; Hodges and Sinclair 2003).

### 2.4 DNA Extraction and 16S rRNA Gene Sequencing

Total DNA was extracted from 50 mg of dried ground faeces and for a negative control using recommended protocols from QIAamp Powerfecal DNA Kit (Cat. 12830, Qiagen) and a bead‐beating step in PowerBead Pro tubes. DNA extracts were quantified using the Quant‐iT PicoGreen Kit (Cat. P7589, Invitrogen) and sent to Genome Quebec (Montreal, Canada) for library preparation and Illumina sequencing (v2 chemistry, MiSeq PE, 250 bp) of the V4 region of the 16S rRNA gene using universal primers 515F and 806R (Walters et al. 2015). Samples used in our analyses were characterised across two separate sequencing batches. Because we detected significant batch effects, we constrained our analyses of seasonal variation to samples from the first sequencing run (70 samples; Table 1), and analyses of cyclical variation in the bacterial microbiome to samples from the second sequencing run (50 samples) to avoid erroneous conclusions from sequencing batch effects.

**TABLE 1:** A summary of the samples used in analyses of variation in the snowshoe hare gut bacterial microbiome between seasons and phases of the population cycle.

Analysis	Cycle phase	Year(s)	Season	Sample size		
				Male	Female	Total
Seasonal	Increase	2013–2015	Summer	16	18	34
		2014–2016	Winter	19	17	36
Cyclical	Increase	2014–2016	Winter	12	3	15
	Peak	2017	Winter	6	6	12
	Decline	2018–2019	Winter	10	13	23

### 2.5 Bioinformatic Processing

Raw paired‐end reads were trimmed, quality filtered and merged using default parameters and a standardised bioinformatics pipeline in the software MOTHUR (Kozich et al. 2013). Briefly, primers were trimmed from sequences and forward and reverse reads were merged into contigs. Where base assignments differed between merged reads, values were assigned to the base of greater quality if the difference in quality scores was ≥ 6 points or an ‘*N*’ if quality scores differed by less than 6 points. Reads with ambiguous base calls or containing homopolymer runs greater than 8 were removed. Screened reads were then aligned against the SILVA reference database trimmed to the same gene region (v138.1; Yilmaz et al. 2014). Sequences failing to overlap this gene region were discarded, and segments of aligned sequences overhanging this gene region were trimmed. To further denoise our dataset, we merged reads which were within 2 nucleotides and retained the most abundant contig as the representative sequence. Chimeric sequences were then identified and removed using VSEARCH (Rognes et al. 2016). Retained contigs were grouped into 97% similarity operational taxonomic units (OTUs) using the OptiClust algorithm (Kozich et al. 2013; Westcott and Schloss 2017). In instances where OTUs were comprised of more than three sequence variants, the sequence with the lowest average distance from other variants was used as a representative sequence for relaxed neighbour joining tree construction using default implementation of the programme CLEARCUT (Sheneman, Evans, and Foster 2006). OTU taxonomy was assigned by using a naïve Bayesian classifier (Wang et al. 2007) and an 80% confidence threshold to map representative sequences against the SILVA ribosomal RNA reference database (v138.1; Yilmaz et al. 2014) to the finest possible level in MOTHUR (Kozich et al. 2013). We generated 6,618,224 read pairs from 120 samples after quality control filtering and merging of read pairs (mean = 55,152, min = 31,703, max = 110,481 reads per sample). Prior to further analyses, we removed OTUs which were only observed in a single sample, resulting in a retention of 6250 OTUs.

### 2.6 Statistical Analyses

#### 2.6.1 Alpha Diversity Analyses

The R (v4.2.1) packages phyloseq (McMurdie and Holmes 2013) and vegan (Oksanen et al. 2008) were used to estimate and analyse patterns of alpha and beta diversity in the bacterial microbiome, unless otherwise stated. We estimated the observed number of OTUs in a sample (OTU richness), Shannon diversity and Faith's phylogenetic diversity as measures of alpha diversity in the gut bacterial microbiome, using datasets rarefied to the lowest sequencing depth (season analyses: 31,550 read/sample; population phase analyses: 43,379 reads/sample). To characterise patterns in alpha diversity, we used general linear models to model alpha diversity measures as a response to sex and the season of sample collection (winter vs. summer), or phase of the population cycle (increase, peak and decline) among samples collected in the winter. Estimates of Faith's phylogenetic diversity were square root transformed in analyses to achieve model assumptions of normality.

To test whether measures of host physiology or faecal fibre contents accounted for observed differences in alpha diversity across phases of the population cycle, we used a multi‐model inference approach using the R package MuMIn (Bartoń 2009). Briefly, we parameterized a global linear model comprised of fixed effects for neutrophil:lymphocyte ratio, mean haematocrit, glucose area under the curve from hormonal challenges, faecal cortisol metabolites, sex and percent faecal fibre content, after confirming that variance inflation factors were < 2. We fitted all possible model fixed effects combinations, compared models using Akaike Information Criterion analysis with a correction applied for small sample sizes (AIC_c_), and estimated parameters using model averaging of all models with a △AIC_c_ < 3. Conditionally averaged parameter estimates were used to determine the statistical significance of effects. The AIC of the top model from multi‐model analyses were compared to that of a model containing only phase of the population cycle.

#### 2.6.2 Beta Diversity Analyses

We calculated Aitchison distances (Euclidean distances of unrarefied centred log ratio‐transformed read count data), as well as UniFrac and weighted UniFrac distances from rarefied datasets, as phylogeny unweighted and weighted measures of bacterial microbiome beta diversity, respectively. Permutational multivariate analysis of variance (PERMANOVA; ‘adonis2’ from R package vegan, by = ‘margin’) tests were used to characterise the marginal effects of sex and season (summer or winter) on bacterial microbiome composition. Similarly, to identify host factors that covaried with bacterial microbiome beta diversity across population phases, we used a PERMANOVA to quantify the marginal effects of faecal fibre content (as a proxy for diet quality) and physiological measures (mean blood haematocrit, N:L ratio, faecal cortisol metabolite concentration, blood glucose) on bacterial microbiome beta diversity. To determine whether hare bacterial microbiomes differed across the population cycle after controlling for significant explanatory factors identified in the preceding analyses, we used additional PERMANOVAs which included a term for phase. We then used post hoc pairwise PERMANOVAs to identify which pairs of phases significantly differed in bacterial microbiome beta diversity. Similarly, post hoc Tukey honest significant difference tests were used to test for multivariate homogeneity of group dispersions between phases of the cycle (‘betadisper’ from R package vegan; measured from group centroids with a bias adjustment for sample size; Anderson, Ellingsen, and McArdle 2006; Oksanen et al. 2008).

To better visualise relative associations of physiological measures or faecal fibre content with the bacterial microbiome and determine whether these measures could underlie phased‐based patterns, we generated canonical analysis of principal coordinates (CAP) biplots constrained by significant non‐phase predictors of microbiome beta diversity identified by PERMANOVA tests. To quantify axes of variation that most cleanly discriminated between phases of the population cycle, we similarly generated a CAP biplot constrained only by the phase in which samples were collected.

#### 2.6.3 Differential Abundance Analyses

To identify bacteria that contributed to compositional differences in the bacterial microbiome between groups (season or phase) we used analyses of compositions of microbiomes with bias correction (ANCOM‐BC) tests (‘ANCOMBC’ R package with Benjamini‐Hochberg corrections for multiple comparisons; Lin and Peddada 2020), including sex as a covariate. We constrained our analyses to the level of bacterial family (the finest taxonomic resolution to which 90% of OTUs could be classified, compared to 45% classification to the level of genus), or alternately for OTUs not classifiable to the level of family, the finest classifiable grouping. We further sought to identify whether compositional differences in the bacterial microbiome across phases were more strongly attributable to faecal fibre contents or significant physiological predictors of beta‐diversity, by completing an additional ANCOM‐BC, parameterized by the factors identified by PERMANOVAs as significant predictors of beta diversity. Among samples collected in the winter, a third ANCOM‐BC test was performed to identify bacterial microbiota that differed between phases, after controlling for physiological factors and faecal fibre content. Correlation tests (Pearson's product‐moment correlations using the cor.test() function from the R package ‘stats’; R Core Team 2024) were used to describe patterns of (dis)similarities between phase‐based differences in family abundances, and family responses to measures of host physiology or faecal fibre contents.

## 3 Results

### 3.1 Bacterial Microbiome Overview

We observed a total of 6250 OTUs (97% cutoff) across a rarefied dataset of 120 snowshoe hare faecal bacterial microbiome samples collected from unique individuals. Mean OTU richness was 342 OTUs ± 94 SD, and the families in the snowshoe hare bacterial microbiome with the highest average relative abundance included Ruminococcaceae (16% ± 7% SD of reads), Prevotellaceae (15% ± 8%), Lachnospiraceae (15% ± 5%), Oscillospiraceae (10% ± 5%), Christensenellaceae (8% ± 5%) and Tannerellaceae (4% ± 2%).

### 3.2 Seasonal Variation in the Bacterial Microbiome

As we predicted, OTU richness (*β* = −52.68 ± 14.54 SE, *t* = −3.62, *p* = 5.6e^−4^; Figure S2a) and square root transformed Faith's phylogenetic diversity (*β* = −0.32 ± 0.08 SE, *t* = −4.20, *p* = 8.1e^−5^; Figure S2b) in the snowshoe hare bacterial microbiome was lower in the winter than in the summer among samples collected during the increase phase of the population cycle (July 2013, 2014, 2015 vs. February 2014, 2015, 2016). However, no difference in Shannon diversity was observed between winter versus summer (*β* = 0.05 ± 0.05 SE, *t* = 0.84, *p* = 0.40; Figure S2c). Males and females likewise did not differ in any measure of alpha diversity (Table S1).

**FIGURE 2:**
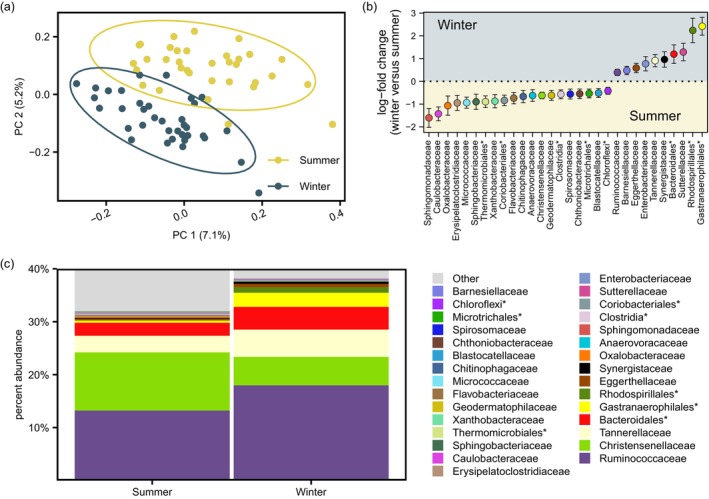
(a) A principal coordinate analysis ordination of Aitchison distances in the snowshoe hare gut bacterial microbiome (OTU‐level) between 70 faecal samples collected in the summer versus winter. (b) Estimates of log‐fold change in bacterial taxon abundance (±standard error) for bacteria (family‐level groupings or coarser) that significantly differed in abundance between the summer and winter (lower yellow panel denotes microbiota enriched in the summer; upper blue panel denotes microbiota enriched in the winter; *Denotes groupings of OTUs which could not be classified to the level of family). (c) A season‐averaged percent abundance bar plot highlighting the bacterial microbiota that differed in abundance between seasons.

Among PERMANOVAs, community composition of the snowshoe hare gut bacterial microbiome differed between winter and summer, across Aitchison (*R* ^2^ = 0.05, *F*_1,67_ = 3.82, *p* < 0.01; Figure 2A), UniFrac (*R* ^2^ = 0.06, *F*_1,67_ = 4.15, *p* < 0.01; Figure S3a), and weighted UniFrac (*R* ^2^ = 0.11, *F*_1,67_ = 8.13, *p* < 0.01; Figure S3b) measurements of beta diversity. Weighted UniFrac beta diversity differed between males and females (*R* ^2^ = 0.03, *F*_1,67_ = 2.02, *p* = 0.03), but no sex effects were observed with respect to Aitchison (*R* ^2^ = 0.02, *F*_1,67_ = 1.09, *p* = 0.21) or UniFrac (*R* ^2^ = 0.02, *F*_1,67_ = 1.16, *p* = 0.11) distances. ANOVA testing of multivariate homogeneity of group dispersions further indicated that bacterial microbiome composition tended to be more variable in summer than winter among UniFrac estimates (estimate = 0.02, 95% CI = 0.001–0.040, *p* = 0.04), but not Aitchison (estimate = −2.29, 95% CI = −4.83–0.25, *p* = 0.08) or weighted UniFrac (estimate = −0.01, 95% CI = −0.03–0.003, *p* = 0.10) estimates of beta diversity.

Of 95 bacterial taxa (family or groupings at higher taxonomic levels) analysed using ANCOM‐BC differential abundance tests, 30 differed in abundance between samples collected in summer versus winter (Figure 2B). Ten taxa were more abundant in winter than summer, including Sutterellaceae, Synergistaceae, Tannerellaceae, Enterobacteriaceae, Eggerthellaceae, Barnesiellaceae and Ruminococcaceae, in addition to bacteria belonging to the orders Gastranaerophilales, Rhodospirillales, Bacteroidales, but which could not be classified to family. Conversely, 20 taxa were less abundant in winter than summer, including Sphingomonadaceae, Caulobacteraceae, Oxalobacteraceae, Erysipelatoclostridiaceae, Micrococcaceae, Sphingobacteriaceae, Xanthobacteraceae, Flavobacteriaceae, Chitinophagaceae, Anaerovoracaceae, Christensenellaceae, Geodermatophilaceae, Spirosomaceae, Chthoniobacteraceae and Blastocatellaceae, in addition to unclassified JG30‐KF‐CM45 Thermomicrobiales, Coriobacteriales, Clostridia, Microtrichales and KD4‐96 Chloroflexi bacteria (Appendix Table S1). Bacteria that differed in abundance between the winter and summer cumulatively comprised 35% of the bacterial microbiome (Figure 2c), on average across 70 individuals sampled (Figure S4).

### 3.3 Cyclical Variation in the Bacterial Microbiome

OTU richness was lower in the peak phase of the population cycle than in either the increase (*β* = −75.69 ± 36 SE, *t* = −2.12, *p* = 0.04; Figure S5a) or decline (*β* = −79.0 ± 32.00 SE, *t* = −2.47, *p* = 0.02) phases, but no difference in richness was observed between the increase and decline phases (*β* = 3.256 ± 31.33 SE, *t* = 0.10, *p* = 0.92). Faith's phylogenetic diversity (square root transformed) was similarly lower in the peak compared to the increase phase (*β* = −0.28 ± 0.13 SE, *t* = −2.15, *p* = 0.04; Figure S5b), but not the decline phase (*β* = −20.0 ± 0.12 SE, *t* = −1.69, *p* = 0.10). No difference in Faith's diversity was observed between the increase and decline phase (*β* = 0.08 ± 0.11 SE, *t* = 0.71, *p* = 0.48). No difference in Shannon diversity was observed between phases of the population cycle, and estimates of alpha diversity did not differ between sexes (Table S2, Figure S5c).

**FIGURE 3:**
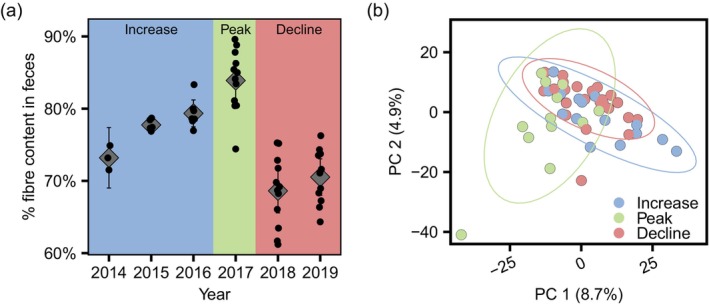
(a) A plot of mean percent fibre content within faecal samples (±95% confidence intervals) collected during each February from 2014 to 2019. Circular points represent individual samples. Panel colours represent phase of the population cycle (blue denotes the increase; green the peak; red the decline). (b) A principal coordinate ordination of OTU level Aitchison distance in the snowshoe hare faecal bacterial microbiome (*n* = 50) coloured by phase of the hare population cycle. Ellipses denote 95% confidence intervals.

To test competing hypotheses that diet versus physiological change underlies differences in bacterial microbiomes across phases of the population cycle we completed multi‐model inference comparisons which modelled alpha diversity measures as a response to combinations of diet quality (faecal fibre content) and physiological (sex, N:L ratio, faecal cortisol metabolite concentration, blood glucose, haematocrit) measures. Despite differences in physiological measures (Lavergne et al. 2021; Figure S6a,b), as well as faecal fibre content within a Tukey's honest significance test (peak > increase > decline: Figure 3a, Table S3) between population phases, no factors were significant predictors of alpha diversity among conditionally averaged models (Table S4). Null models among multi‐model comparisons differed only superficially from the top models explaining variation in OTU richness (faecal fibre content term only: △AIC_c_ = 0.17) and Faith's phylogenetic diversity (blood glucose term only: △AIC_c_ = 0.35) or performed worse than the null model in the instance of Shannon diversity.

Aitchison and UniFrac measures of faecal bacterial microbiome beta diversity varied in response to hare sex, faecal cortisol metabolite concentration, and faecal fibre content, among PERMANOVA tests for marginal effects (Table S5). Aitchison (but not UniFrac) measurements of beta diversity also differed in response to mean haematocrit estimates. Weighted UniFrac measures of beta diversity varied with faecal fibre content, but did not differ between sexes or in response to physiological measures (Table S5).

**FIGURE 4:**
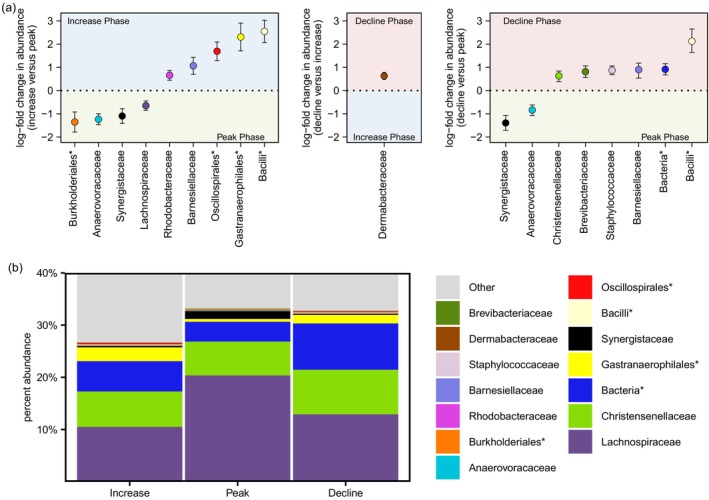
(a) Estimates of log‐fold change in taxon abundance (±standard error) for bacteria that significantly differed in abundance across 50 snowshoe hare bacterial microbiome samples, between phases of the population cycle (blue panel denotes relative enrichment in the increase; green panel denotes relative enrichment in the peak; red panel denotes relative enrichment in the decline; *Denotes groupings of OTUs which could not be classified to family). (b) A phase‐averaged percent abundance bar plot highlighting the bacterial microbiota that differed in abundance between phases.

Principal coordinate analysis (PCoA) ordinations of Aitchison (Figure 4A), UniFrac (Figure S7a), and weighted UniFrac (Figure S7b) beta diversity evidenced phased‐based differences in gut bacterial communities. Even after controlling for physiological measures and faecal fibre content, phased‐based differences in the bacterial microbiome were identified by PERMANOVA tests of Aitchison (*R* ^2^ = 0.05, *F*_2,34_ = 1.31, *p* < 0.01), UniFrac (*R* ^2^ = 0.05, *F*_2,34_ = 1.31, *p* = 0.001) and weighted UniFrac (*R* ^2^ = 0.08, *F*_2,34_ = 2.00, *p* < 0.01) distances. Post hoc pairwise PERMANOVA tests indicated that phase‐based differences in the bacterial microbiome were attributable to differences between the peak versus the increase or decline phases. We detected no differences in beta diversity between the increase and decline phases. We did not observe evidence for differences in bacterial community variability between phases, based upon tests of multivariate homogeneity of group dispersions (Table S6 and S7).

ANCOM‐BC differential abundance tests identified five bacterial groupings at the family level or higher which were more abundant in the increase phase than peak phase (Barnesiellaceae, Rhodobacteraceae, and unclassified Gastranaerophilales, UCG‐010 Oscillospirales and RF39 Bacilli; Figure 4a, Appendix Table S2), while four taxa were more abundant in the peak than increase phase (Anaerovoracaceae, Synergistaceae, Lachnospiraceae and unclassified Burkholderiales). Six taxa were more abundant in the decline than peak phase (Barnesiellaceae, Staphylococcaceae, Brevibacteriaceae, Christensenellaceae, unclassified RF39 Bacilli and a collection of unclassified bacterial OTUs), while only two families were more abundant in the peak than decline phase (Anaerovoracaceae and Synergistaceae). Only a single family (Dermabacteraceae) was more abundant, and no bacteria were less abundant, in the decline than increase phase. Bacteria which significantly differed between phases of the population cycle cumulatively represented 31% of bacterial reads (Figure 4b), on average across 50 sampled individuals (Figure S8).

**FIGURE 5:**
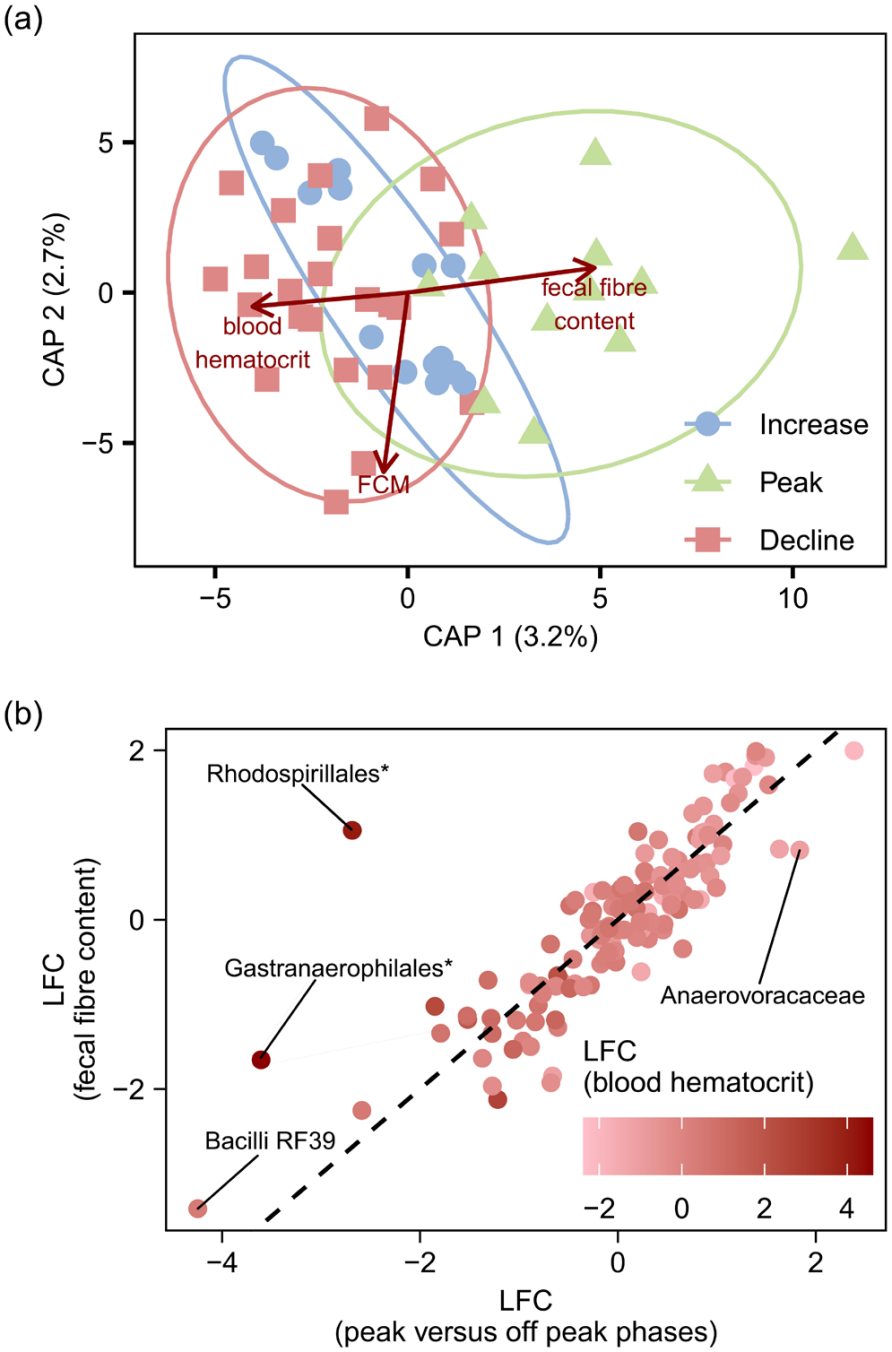
(a) A canonical analysis of principal coordinates plot of OTU level Aitchison distances constrained by faecal fibre content, blood haematocrit, and faecal cortisol metabolite concentration. Points represent individual samples (*n* = 50), ellipses represent 95% confidence intervals, and arrows represent loadings of constraint variables. (b) Analysis of composition of microbiomes test estimates of log fold change (LFC; centred and variance standardised) in bacterial taxon (family or coarser) abundance between peak and off‐peak phases of the hare population cycle versus bacterial associations with faecal fibre content, coloured by associations with mean haematocrit. Dotted diagonal denotes the 1:1 line.

A canonical analysis of principal coordinates (CAP) biplot of Aitchison distance constrained by significant predictors of beta diversity (mean haematocrit, faecal cortisol metabolite concentration and faecal fibre content) evidenced separation between population phases (Figure 5a). Specifically, samples collected in the peak phase separated from those collected during the increase and decline phases along the same axis of variation that paralleled biplot vectors for faecal fibre content (CAP 1 loading = 0.81, CAP 2 loading = 0.14) and mean haematocrit (CAP 1 loading = −0.67, CAP 2 loading = −0.08). In contrast, the vector describing faecal cortisol metabolite concentration loaded orthogonally to the axis which most clearly separated samples collected during the peak phase, from those in the increase or decline phases (CAP 1 loading = −0.10, CAP 2 loading = −0.99; Figure 5a).

ANCOM‐BC tests identified five bacterial taxa that were significantly positively associated with faecal fibre content, including Synergistaceae, Micromonosporaceae and unclassified Gitt‐GS‐136 Chloroflexi, Vicinamibacterales, and JG30‐KF‐CM45 Thermomicrobiales, and seven taxa negatively associated with faecal fibre content, including Desulfovibrionaceae, Brevibacteriaceae, Oscillospiraceae, Staphylococcaceae, Christensenellaceae and unclassified UCG‐010 Oscillospirales, and a grouping of bacterial OTUs which could not be classified to phylum. Neither mean haematocrit nor faecal cortisol metabolite concentrations were significantly associated with any bacterial taxon, after false discovery bias correction for multiple comparisons (Appendix Table S3).

We next pooled samples from the increase and decline phases of the population cycle, because we observed only marginal differences in bacterial microbiome composition between these phases (see above). Only two families continued to differ in abundance between peak and off‐peak phases, within ANCOM‐BC tests, after controlling for mean haematocrit, faecal cortisol concentration, sex and faecal fibre content. Anaerovoracaceae was more abundant, but the RF39 clade of Bacilli was less abundant, among samples collected in the peak phase when compared to samples collected during off‐peak phases (Appendix Table S4). Estimates of log‐fold differences in bacterial taxon abundance in association with faecal fibre content strongly covaried with differences in abundance between peak and off‐peak phases, with the notable exception of Gastranaerophilales and Rhodospirillales (Pearson's product‐moment correlation: *r* = 0.84, *t* = 17.14, *p* = 2.2e^−16^; Figure 5b). Differences in bacterial taxon abundances between peak and off‐peak phases were more weakly correlated with relationships to blood haematocrit (*r* = −0.66, *t* = −9.91, *p* = 2.2e^−16^; Figure S9a), but not faecal cortisol metabolites (*r* = 0.004, *t* = 0.04, *p* = 0.97; Figure S9b). Relationships between unclassified Gastranaerophilales, as well as Rhodospirillales, and blood haematocrit accounted for the outlying responses of these bacteria to faecal fibre content (Figure 5b).

**FIGURE 6:**
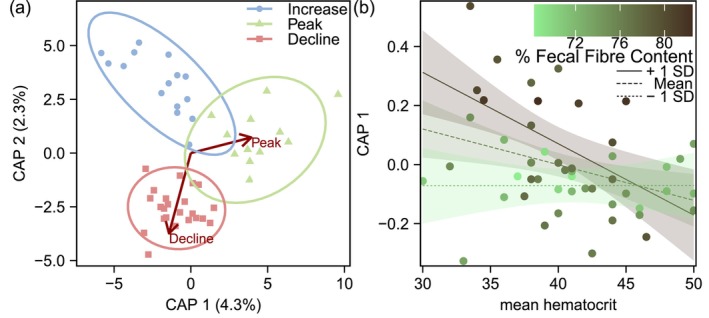
(a) A canonical analysis of principal coordinates plot of OTU level Aitchison distances in the hare faecal bacterial microbiome constrained by phase of the population cycle. Points represent individual samples (*n* = 50), ellipses represent 95% confidence intervals, and arrows represent loadings of constraint variables. (b) An interaction plot illustrating the modulating effect of percent faecal fibre content on the relationship between blood haematocrit, and the first canonical analysis of principal coordinates axis (CAP 1 from panel ‘a’) which distinguishes faecal bacterial microbiome differences between peak versus off‐peak phases of the hare population cycle. Shading denotes 95% confidence intervals. Lines represent best fit relationships under conditions of mean, or at 1 standard deviation extremes, in faecal fibre content.

A lack of difference in the microbiome between the decline and increase phases (despite large differences in faecal fibre content), and the findings that haematocrit better accounted for differences in Rhodospirillales and Gastranaerophilales abundance between peak and off‐peak phases led us to propose the post hoc hypothesis that interactions between diet quality (faecal fibre content) and host condition (blood haematocrit) could explain differences in the bacterial microbiome between peak versus off peak phases of the population cycle. To test this hypothesis, we first generated a canonical analysis of principal coordinates ordination constrained by population cycle phase, to obtain an axis of bacterial microbiome variation (CAP 1) that discriminates between peak and off‐peak phases (Figure 6a). In testing our hypothesis using a general linear model, we observed that CAP 1 values are significantly associated with faecal fibre content (*β* = 0.08 ± 0.02 SE, *t* = −3.16, *p* = 3.0^−3^), haematocrit (*β* = 0.12 ± 0.05 SE, *t* = 2.34, *p* = 0.01), and an interaction between these two terms (*β* = −0.002 ± 0.0006 SE, *t* = −2.75, *p* < 0.001), whereby a negative relationship between blood haematocrit and CAP 1 was greatest at high levels of faecal fibre content (Figure 6b).

## 4 Discussion

Snowshoe hare populations in the boreal forests of North America experience two notable recurring environmental challenges: (1) seasonality, and (2) decadal boom‐bust population cycles characterised by fluctuations in hare density, predation risk, diet and shifting anti‐herbivory PSM exposure (Boonstra et al. 1998; Boutin et al. 1986; Deangelis et al. 2015; Hodges, Boonstra, and Krebs 2006; Krebs, Boonstra et al. 2001; Krebs, Dale et al. 2001; Sheriff, Krebs, and Boonstra 2009, 2010; Sinclair, Krebs, and Smith 1982; Sinclair et al. 1988; Smith et al. 1988). In characterising the responses of hare faecal bacterial microbiomes to these challenges, we found marked differences in bacterial communities between seasonal extremes of summer and winter, and to a subtler extent, between phases of the population cycle among samples collected in winter.

Winter‐collected faecal samples exhibited lower bacterial OTU richness and phylogenetic diversity, when compared to summer samples. We speculate these, and differences in community composition, are partly attributable to dietary differences between seasons, as hare diets are more diverse in the summer than winter (Seccombe‐Hett and Turkington 2008), which could support a greater breadth of metabolic niches in the gut environment (Pereira and Berry 2017; Reese and Dunn 2018). Similar seasonal restructuring of the gut microbiome has been observed in other wildlife between summer and winter at high latitudes or altitudes (Bird et al. 2019; Carey, Walters, and Knight 2013; Marsh et al. 2022; Stothart et al. 2023) and between wet and dry seasons at low latitudes (Björk et al. 2022; Klure and Dearing 2023; Orkin et al. 2019). Alongside diet, exposure to a differing pool of environmental microbiota in the summer than winter may contribute to these patterns (Uroz et al. 2016). For example, local variation in soil properties can cause differences in gut microbiota communities between animal populations (Grieneisen et al. 2019), and the effects of soil microbiomes on bacterial communities in the animal gut might be especially strong for soil‐consuming (geophagic) species, like snowshoe hare (Rea, Stumpf, and Hodder 2013; Worker, Kielland, and Barboza 2015). However, since summer samples were collected from the ground below traps, we cannot discount the possibility that seasonal differences were partly attributable to contamination by soil.

Variation in the faecal bacterial microbiome across winters of the snowshoe hare population cycle were primarily attributable to differences between the peak versus off‐peak (increase and decline) phases. Bacterial microbiomes during the peak phase of the population cycle were characterised by lower OTU richness and compositional differences, when compared with faeces sampled during the increase and decline phases (which were indistinguishable in both alpha and beta diversity). These results do not support the hypothesis that population phase‐based changes in the microbiome are caused by predation‐related stress. First, predation stressors are greatest during the decline phase and lowest in the increase phase of the population cycle (Boonstra et al. 1998; Sheriff, Krebs, and Boonstra 2009, 2010). Second, FCMs were only weakly associated with compositional variation in the bacterial microbiome, not significantly associated with any bacterial taxon tested, and the community variation explained by FCM was orthogonal to phase‐based variation among constrained ordination biplots.

Our findings more strongly support the alternative hypothesis that phase‐based differences in the bacterial microbiome are related to changes in diet. Faecal fibre contents paralleled hare population density (highest in the peak phase, lowest in the decline) and relationships of bacterial microbiota abundances to faecal fibre contents were correlated with differences observed between peak versus off‐peak phases of the population cycle, although this does necessarily signify a causal relationship. However, despite the explanatory power of faecal fibre content, this measure did not entirely account for compositional differences in the bacterial microbiome between phases among PERMANOVA tests, nor phase‐based differences in Anaerovoracaceae, Bacilli RF39, Gastranaerophilales or Rhodospirillales abundance. Furthermore, no compositional differences in the bacterial microbiome were observed between the increase and decline phases, despite greater differences in faecal fibre content between these phases (△8%), when compared to between the peak and increase phases (△6%). The absence of bacterial microbiome differences between increase and decline phases, despite differences in faecal fibre content, could reflect non‐linear relationships between the bacterial microbiome and dietary fibre or modulating effects by other diet or physiological factors, as evidenced by our observation of faecal fibre interactions with blood haematocrit.

The ecology of the bacterial families which differed in abundance between seasons and phases provide inferential support for an interpretation of diet as a causal mechanism. For example, the two most abundant bacterial families to differ seasonally included Ruminococcaceae (enriched in winter) and Christensenellaceae (enriched in summer). The family Ruminococcaceae contains important plant fibre degrading bacteria common in the herbivorous mammalian gut (Biddle et al. 2013; Flint et al. 2008; Julliand et al. 1999; Rainey 2009; Ren et al. 2017; Stewart et al. 2019), while many Christensenellaceae are associated with bile acid metabolism, monosaccharide fermentation, and a labile diet (Morotomi, Nagai, and Watanabe 2011; Ignatyeva et al. 2024; Stothart, McLoughlin, and Poissant 2023; Waters and Ley 2019). Seasonal shifts in these families could reflect that, while hares have access to labile leaf material in the summer (Seccombe‐Hett and Turkington 2008), during the winter hares become increasingly reliant upon fibrous and lignin‐rich foods ( *Betula glandulosa* and *Salix glauca* , *Picea glauca* twigs; Sinclair et al. 1988; Smith et al. 1988; Wolff 1978).

The association of Christensenellaceae with a labile diet might similarly explain enrichment of this family in the decline compared to peak phases, between which we observed the greatest differences in faecal fibre content. Despite differences in faecal fibre content between population phases, we observed no difference in Ruminococcaceae abundance between population cycle phases, nor evidence for a relationship between Ruminococcaceae abundance and faecal fibre content. Three samples collected during the peak phase showed apparent depletion of Ruminoccocaceae which could signal a diseased microbiome state; but notably, phase‐based differences in the microbiome persisted even after removal of these outliers (Figure S10). Although fibre can be an important dietary determinant of the gut microbiome, other plant compounds likely contribute to both seasonal and phase‐based variation in the snowshoe hare microbiome.

The leaves, roots, and fruits of many deciduous plants contain oxalate, an anti‐nutritious compound that can be degraded by some Oxalobacteraceae (Miller et al. 2016), a bacterial family that was more abundant in hare faeces during the summer than winter. Food resources used by hares in the winter are not only fibrous, but rich in non‐oxalate PSMs (Deangelis et al. 2015; Sinclair and Smith 1984). Ingestion of more PSMs by hares during winter might explain greater abundance of Synergistaceae in winter than summer, since some Synergistaceae metabolise PSMs in the herbivore gut (Allison et al. 1992; Kang et al. 2020) and are even thought to be key detoxifiers of a Eucalyptus‐rich diet in koala, *Phascolarctos cinereus* (Blyton et al. 2019; Shiffman et al. 2017).

Alongside seasonal shifts in abundance, Synergistaceae exhibited the strongest positive relationship with faecal fibre content of all bacterial microbiota tested and were more abundant in the peak phase when compared with either the increase or decline phases. Preferred food resources (small stem birch and willow) are diminished as snowshoe hare populations approach the peak phase of their population cycle, and hares become increasingly reliant on less preferred, poor quality, large diameter twigs (Krebs, Dale et al. 2001; Smith et al. 1988). However, if Synergistaceae are involved in PSM metabolism as we hypothesise, then its elevation during the peak phase runs counter to our initial predictions, since PSM concentrations in preferred food species are highest in the decline phase (Bryant et al. 1985; Deangelis et al. 2015). Depletion of these food resources can shift hares onto more heavily PSM‐defended forage; for example, young white spruce twigs and needles (Sinclair and Smith 1984; Smith et al. 1988; Wolff 1978) which are rich in the PSM camphor (Sinclair, Jogia, and Andersen 1988). Synergistaceae enrichment during the peak phase could reflect a dietary transition towards high fibre and high camphor spruce, as birch and willow food resources become depleted.

Metagenomic characterisation of the hare microbiome is an important next step, since short‐read amplicons offer limited taxonomic resolution and no direct measures of gene content (Stothart, McLoughlin, and Poissant 2023). For example, most reads obtained from the hare gut could not be classified beyond family, and yet many functional traits, including those for cellulose or simple carbon substrate utilisation, are shallowly conserved at the tips of bacterial phylogenies, rather than deeply rooted traits (Martiny et al. 2015). Since we did not directly measure gene content or transcriptional differences between seasons or phases, our functional inferences should be interpreted as hypotheses rather than conclusions. Finally, while we detected both seasonal and phase‐based variation in the hare microbiome, our small winter sample size may have been insufficient to identify subtler physiological determinants of bacterial microbiome variation. Microbiota responses to physiological measures may also occur at the level of OTU (rather than family), but our low sample size precluded us from testing this hypothesis, as this would require a greater number of tests and a more conservative false discovery rate bias correction. Nonetheless, our observation of strong faecal fibre contents, despite a low sample size, provides insight as to the relative strength of dietary versus physiological effects on the bacterial microbiome among snowshoe hares.

Bacterial communities in the snowshoe hare gut differed between summer and winter, and among winters, across boom‐bust phases of the decadal hare population cycle. Seasonal effects were stronger than those of cycle phase, but differentiation of the gut microbiome in the peak phase of the population cycle nonetheless offers support for suggestions that microbiomes might be used as bioindicators of wildlife health (Ribas et al. 2023), especially when population declines are coincident with changes in the food resources. Direct measures of host physiology and faecal fibre content most strongly support a microbiome connection to a changing dietary landscape across phases of the population cycle, but with possible modulating effects of host condition. Further experimental research is needed to determine if phase‐based microbiome differences are caused by, non‐causally coincident with, or occur in anticipation of, changes in food resource availability. Beyond characterising the causes of bacterial community variation between seasons and across phases, an important next step is to determine the fitness consequences of bacterial microbiome variation within years, particularly during peak phases of the population cycle when selective pressures on the microbiome might be strongest. This study represents the first step towards understanding the eco‐evolutionary significance of gut microbiome variation within this keystone species and intensively studied population in the boreal forest ecosystem.

## Author Contributions

Project conception by Sophia Lavergne with input on study design from Katherine Amato, Wilfred de Vega and Mason R. Stothart. Animal handling and sampling by Sophia Lavergne and Rudy Boonstra. Sample preparation and DNA extractions by Sophia Lavergne, Hardeep Singh and Laura McCaw. Fibre assays by Laura McCaw. Bioinformatic and statistical analyses by Mason R. Stothart. Original manuscript written by Sophia Lavergne, Mason R. Stothart and Rudy Boonstra. All authors read, revised and approved the final manuscript.

## Ethics Statement

All handling and study procedures were approved by the Yukon Territorial Government (Scientists and Explorers Act Licences and Wildlife Permits, 2013–2019), as well as by the University of Toronto Animal Care Committee in accordance with the guidelines of the Canadian Council for Animal Care.

## Conflicts of Interest

The authors declare no conflicts of interest.

## Supporting information


Data S1.

**Appendix Table S1.** Statistical output from ANCOM‐BC tests of differential abundances in bacterial families within the snowshoe hare gut, between samples collected in the summer versus winter with Benjamini‐Hochberg corrections for multiple comparisons. Default parameters and a prevalence threshold of 10% applied to tests.
Appendix Table S2. Statistical output from ANCOM‐BC tests of differential abundances in bacterial families within the snowshoe hare gut, across phases of the hare population cycle with Benjamini‐Hochberg corrections for multiple comparisons. Default parameters and a prevalence threshold of 10% applied to tests.
Appendix Table S3. Statistical output from ANCOM‐BC tests of differential abundances in bacterial families within the snowshoe hare gut, in response to faecal fibre content, mean haematocrit measures, faecal cortisol metabolite concentration and sex, among samples collected in the winter across phases of the lynx‐hare population cycle with Benjamini‐Hochberg corrections for multiple comparisons. Default parameters and a prevalence threshold of 10% applied to tests.
Appendix Table S4. Statistical output from ANCOM‐BC tests of differential abundances in bacterial families within the snowshoe hare gut in the peak versus off‐peak (increase versus decline) phases of the hare population cycle, after controlling for faecal fibre content, mean blood haematocrit, faecal cortisol metabolite concentration and sex, among samples collected in the winter with Benjamini‐Hochberg corrections for multiple comparisons. Default parameters and a prevalence threshold of 10% applied to tests.

## Data Availability

Raw sequence reads and sample metadata are deposited in the NCBI SRA (BioProject PRJNA1092460; https://www.ncbi.nlm.nih.gov/bioproject/PRJNA1092460/; Stothart et al. 2024a). Processed sequencing data, metadata and R scripts are available on FigShare (doi: 10.6084/m9.figshare.25540078; Stothart et al. 2024b).
