# Supplementary material for: Population Dynamics and the Microbiome in a Wild Boreal Mammal: The Snowshoe Hare Cycle and Impacts of Diet, Season and Predation Risk
Source: Mol Ecol. 2024 Dec 19;34(3):e17629. doi: 10.1111/mec.17629 (PMC11754720; doi:10.1111/mec.17629)
Supplement: Supplementary file 1 — Data S1. [file MEC-34-e17629-s002.pdf]

MOLECULAR ECOLOGY

Supplemental Information for:

Population dynamics and the microbiome in a wild boreal mammal: The snowshoe hare cycle and impacts of diet, season, and predation risk

Mason Stothart*[1,2], Sophia Lavergne*[3], Laura McCaw[3], Hardeep Singh[3], Wilfred de Vega[3], Katherine Amato[4], Jocelyn Poissant[1], Rudy Boonstra[3]

**Equal contribution by authors*

[1] Faculty of Veterinary Medicine, University of Calgary, Calgary, Canada

[2] Department of Biology, University of Oxford, Oxford, UK

[3] Department of Biological Sciences, University of Toronto Scarborough, Toronto, Canada

[4] Department of Anthropology, Northwestern University, Evanston, USA

Table of Contents:

**Supplementary Table S1**	Page 2
**Supplementary Table S2**	Page 3
**Supplementary Table S3**	Page 4
**Supplementary Table S4**	Page 5
**Supplementary Table S5**	Page 6
**Supplementary Table S6**	Page 7
**Supplementary Table S7**	Page 8
**Supplementary Figure S1**	Page 9
**Supplementary Figure S2**	Page 10
**Supplementary Figure S3**	Page 11
**Supplementary Figure S4**	Page 12
**Supplementary Figure S5**	Page 13
**Supplementary Figure S6**	Page 14
**Supplementary Figure S7**	Page 15
**Supplementary Figure S8**	Page 16
**Supplementary Figure S9**	Page 17
**Supplementary Figure S10**	Page 18
**Supplementary References**	Page 19

MOLECULAR ECOLOGY

Table S1. Statistical outputs from general linear models fitted to measures of alpha diversity (OTU richness, Faith's phylogenetic diversity, Shannon diversity) in 70 snowshoe hare fecal microbiome samples as a response to season (summer versus winter, summer base) and sex (female base). Bolded rows denote statistical significance at $p < 0.05$.

	OTU richness				Faith's phylogenetic diversity†				Shannon diversity			
**Term**	**β**	**SE**	***t***	***p***	**β**	**SE**	***t***	***p***	**β**	**SE**	***t***	***p***
Intercept	**350.39**	**12.46**	**28.125**	**$2e^{-16}$**	**4.46**	**0.06**	**69.100**	**$2e^{-16}$**	**3.50**	**0.05**	**76.712**	**$2e^{-16}$**
Season	**−52.68**	**14.54**	**−3.622**	**$5.6e^{-4}$**	**−0.32**	**0.08**	**−4.200**	**$8.1e^{-5}$**	0.05	0.05	0.844	0.40
Sex	−16.13	14.54	−1.110	0.27	−0.09	0.08	−1.236	0.22	0.008	0.05	0.159	0.87

†Values square root transformed.

MOLECULAR ECOLOGY

Table S2. Statistical outputs from general linear models fitted to measures of alpha diversity (OTU richness, Faith's phylogenetic diversity, Shannon diversity) in 50 snowshoe hare fecal microbiome samples as a response to sex and phase of the hare population cycle (increase, peak, decline). Bolded rows denote statistical significance at $p < 0.05$.

	OTU richness				Faith's phylogenetic diversity†				Shannon diversity			
**Term**	**β**	**SE**	***t***	***p***	**β**	**SE**	***t***	***p***	**β**	**SE**	***t***	***p***
Phase (increase - peak)	**75.69**	**35.64**	**2.124**	**0.04**	**0.28**	**0.13**	**2.145**	**0.04**	0.03	0.13	0.207	0.84
Phase (decline - peak)	**78.95**	**31.96**	**2.470**	**0.02**	0.20	0.12	1.693	0.10	0.01	0.12	0.122	0.90
Phase (decline - increase)	3.256	31.332	0.104	0.92	−0.08	0.11	−0.713	0.48	−0.01	0.11	−0.112	0.91
Sex	11.96	26.96	0.444	0.66	−0.05	0.10	−0.502	0.62	0.08	0.10	0.207	0.84

†Values square root transformed.

MOLECULAR ECOLOGY

Table S3. Statistical outputs from a Tukey's honest significance test of differences in percent fecal fibre content between phases of the snowshoe hare population cycle (increase, peak, decline; 50 samples). Bolded rows denote statistical significance at $p < 0.05$.

**Contrast**	**difference**	**lower**	***upper***	***p-value***
Peak – Increase	**6.32**	**2.62**	**10.02**	**4.2e^{-4}**
Decline – Increase	−8.05	−11.22	−4.89	**<5.0e^{-7}**
Decline – Peak	−14.37	−17.77	−10.97	**<1e^{-8}**

MOLECULAR ECOLOGY

Table S4. Multi-model inference statistical outputs from AIC_c model averaging results ($\triangle AIC_c < 3$ threshold), in which measures of alpha diversity (OTU richness, Faith's phylogenetic diversity, and Shannon diversity) within 50 snowshoe hare fecal bacterial microbiome samples during the winter were modeled as a response to physiological or diet factors (centered and variance standardized). Bolded rows denote statistical significance at $p < 0.05$.

	**OTU richness**				**Faith's phylogenetic diversity†**				**Shannon diversity**			
**Term**	**β**	**SE**	***z***	***p***	**β**	**SE**	***z***	***p***	**β**	**SE**	***z***	***p***
Intercept	**553.42**	**16.31**	**33.214**	**$2e^{-16}$**	**5.17**	**0.05**	**101.52**	**$2e^{-16}$**	**3.30**	**0.05**	**60.303**	**$2e^{-16}$**
Sex (female base)	29.31	28.74	0.996	0.32	0.04	0.10	0.402	0.69	0.07	0.09	0.78	0.43
N:L ratio	−13.19	13.89	0.926	0.355	−0.05	0.05	0.945	0.35	−0.07	0.05	1.408	0.16
Fecal cortisol	−13.46	13.67	0.959	0.34	−0.04	0.05	0.88	0.38	−0.06	0.05	1.218	0.22
Blood glucose AUC	12.87	13.82	0.909	0.36	0.10	0.05	1.89	0.06	0.01	0.05	0.308	0.76
Mean hematocrit	10.15	14.28	0.694	0.49	0.07	0.05	1.349	0.18	0.01	0.05	0.292	0.77
Fecal fibre content	−22.46	13.91	1.575	0.12	−0.03	0.05	0.67	0.50	0.02	0.05	0.344	0.73

†Values square root transformed.

MOLECULAR ECOLOGY

Table S5. Statistical outputs from permutational multivariate analyses of variance of Aitchison, UniFrac, and weighted UniFrac beta diversity in 50 snowshoe hare fecal bacterial microbiome samples during the winter in response to physiological factors or fecal fibre contents. Bolded rows denote statistical significance at $p < 0.05$.

	**Aitchison Distance**			**UniFrac**			**weighted UniFrac**		
**Term**	**R^2**	**F**	***p***	**R^2**	**F**	***p***	**R^2**	**F**	***p***
Sex	**0.03**	**1.402**	**0.01**	**0.03**	**1.341**	**0.01**	0.03	1.615	0.09
N:L ratio	0.02	0.944	0.60	0.02	0.942	0.67	0.03	1.568	0.12
Mean hematocrit	**0.02**	**1.229**	**0.05**	0.02	1.160	0.07	0.02	0.784	0.64
Fecal cortisol	**0.03**	**1.337**	**0.02**	**0.02**	**1.198**	**0.04**	0.03	1.581	0.10
Blood glucose AUC	0.02	1.133	0.13	0.02	1.144	0.09	0.03	1.507	0.13
Fecal fibre content	**0.03**	**1.610**	**<0.01**	**0.03**	**1.510**	**<0.01**	**0.04**	**1.871**	**0.05**

MOLECULAR ECOLOGY

Table S6. Statistical outputs from pairwise permutational multivariate analyses of variance of Aitchison, UniFrac, and weighted UniFrac beta diversity in 50 snowshoe hare fecal bacterial microbiome samples across increase, peak, and decline phases of the population cycle. Bolded rows denote statistical significance at $p < 0.05$.

	**Aitchison Distance**			**UniFrac**			**weighted UniFrac**		
**Term**	**R^2**	**F**	***p***	**R^2**	**F**	***p***	**R^2**	**F**	***p***
Decline versus Increase	0.03	1.178	0.06	0.03	1.117	0.10	0.04	1.536	0.11
Decline versus Peak	**0.05**	**1.893**	**$1.0e^{-4}$**	**0.05**	**1.678**	**$2.0e^{-4}$**	**0.09**	**3.079**	**$2.3e^{-3}$**
Increase versus Peak	**0.07**	**1.972**	**$2.0e^{-4}$**	**0.06**	**1.638**	**$3.0e^{-4}$**	**0.09**	**2.572**	**$4.5e^{-3}$**

MOLECULAR ECOLOGY

Table S7. Statistical outputs from tests of multivariate homogeneity of group dispersions in Aitchison, UniFrac, and weighted UniFrac beta diversity in 50 snowshoe hare fecal bacterial microbiome samples across increase, peak, and decline phases of the population cycle. Bolded rows denote statistical significance at p < 0.05.

	**Aitchison Distance**				**UniFrac**				**weighted UniFrac**			
**Term**	**SS**	**MS**	$F_{2,47}$	***p***	**SS**	**MS**	$F_{2,47}$	***p***	**SS**	**MS**	$F_{2,47}$	***p***
Phase	16.87	8.44	0.364	0.70	0.001	0.0007	0.837	0.44	0.012	0.006	1.836	0.17
Residuals	1088.84	23.17	-	-	0.038	0.0008	-	-	0.152	0.003	-	-

MOLECULAR ECOLOGY

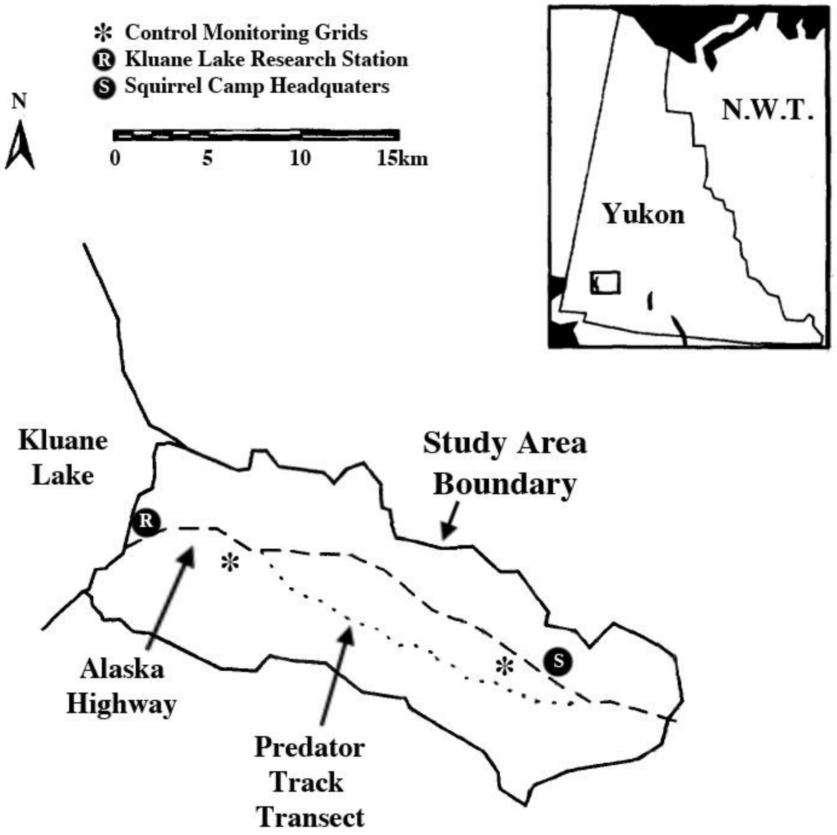

Figure S1: Kluane Lake research study area (Yukon Territory). Trapping sessions targeted hares in the boreal forest surrounding a 30-km stretch of the Alaska Highway between the Arctic Institute of North America’s Kluane Lake Research Station and the Kluane Red Squirrel Project headquarters. CEMP monitoring activities were focused on two control monitoring grids and a 22 km winter track transect. Map adapted from O’Donoghue et al. (1997).

MOLECULAR ECOLOGY

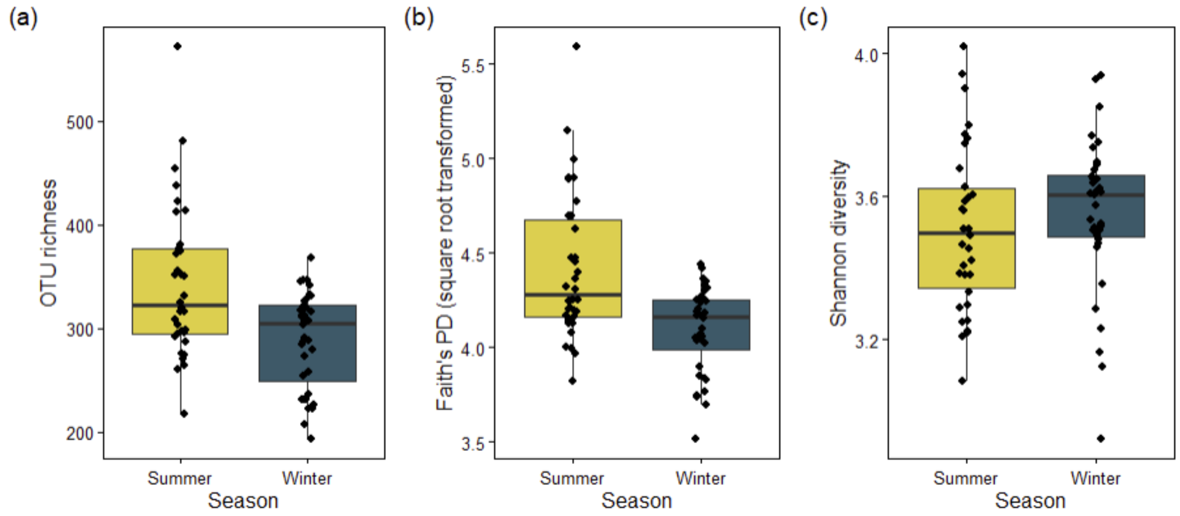

Figure S2. (a) OTU richness, (b) Faith's phylogenetic diversity (square root transformed), and (c) Shannon diversity in the snowshoe hare fecal bacterial microbiome (70 samples) between summer and winter. Bold horizonal lines represent medians, boxes represent the interquartile range (IQR), whiskers represent the Q1 or Q3 ± 1.5 x IQR, and points represent individual samples.

MOLECULAR ECOLOGY

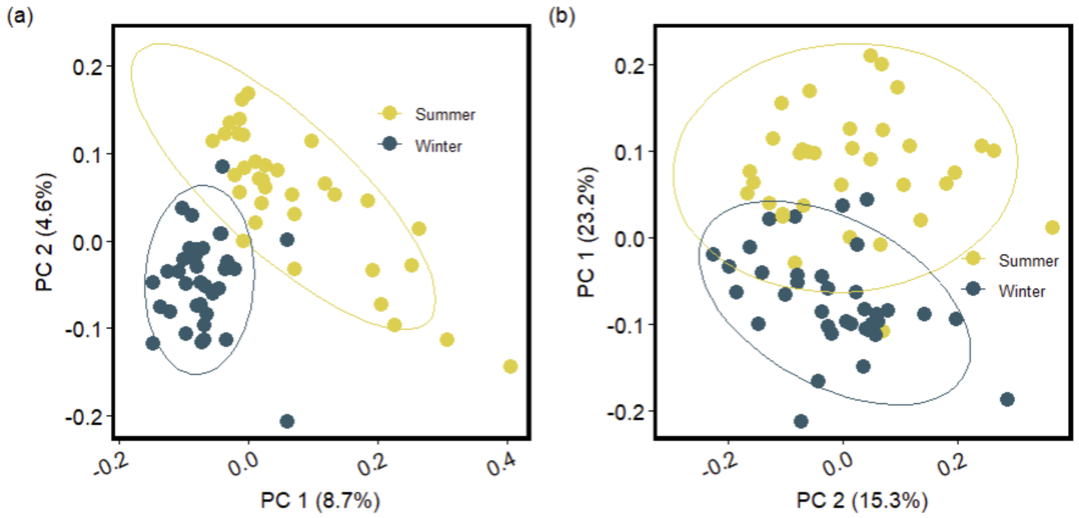

Figure S3. Principal coordinate analysis ordinations of (a) UniFrac and (b) weighted UniFrac dissimilarities in the snowshoe hare fecal bacterial microbiome (OTU-level) between samples collected in the summer versus winter (70 samples). Points represent individual samples and circles denote 95% confidence ellipses.

MOLECULAR ECOLOGY

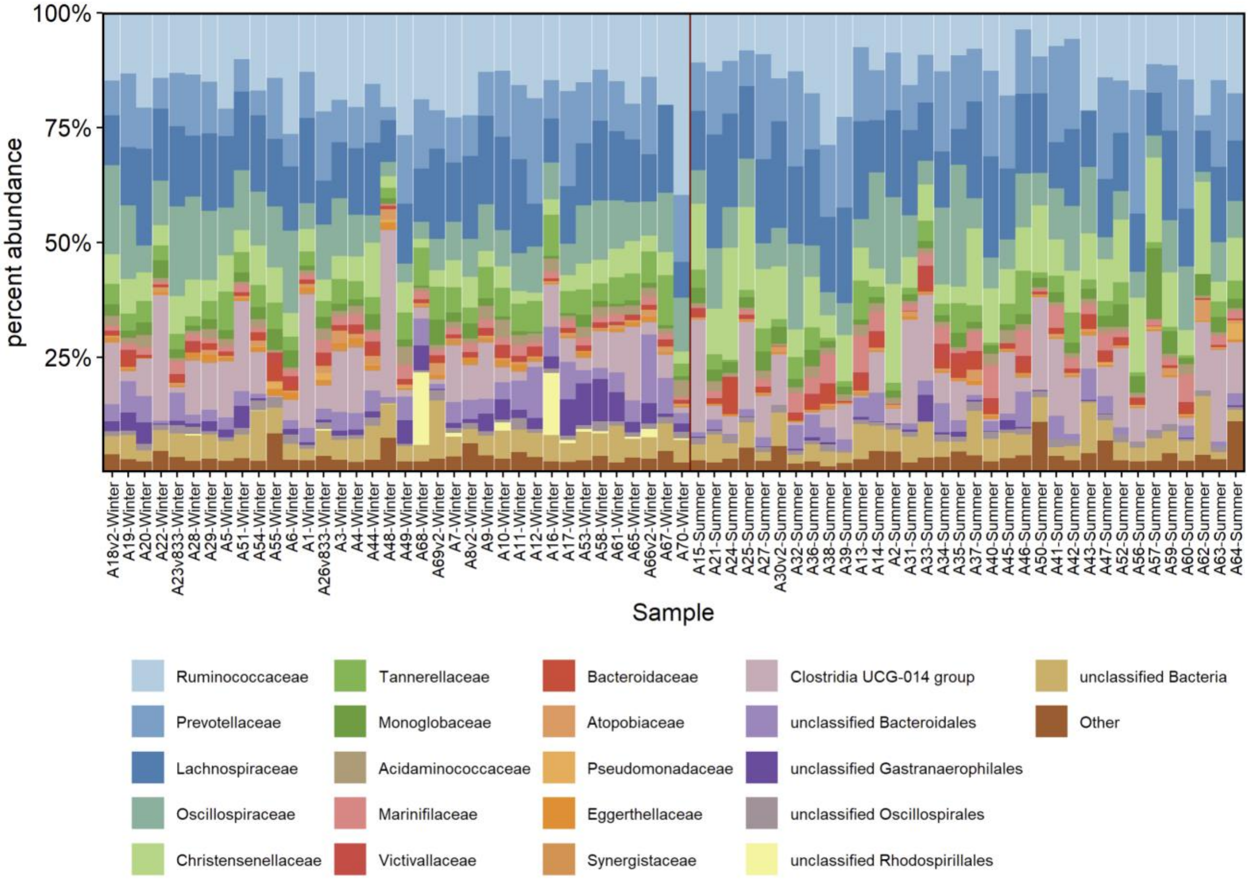

Figure S4. A barplot of bacterial taxa (family level or or coarser) in the snowshoe hare fecal microbiome. Each column represents a different individual (n = 70). Vertical red lines separate samples collected in the summer versus winter.

MOLECULAR ECOLOGY

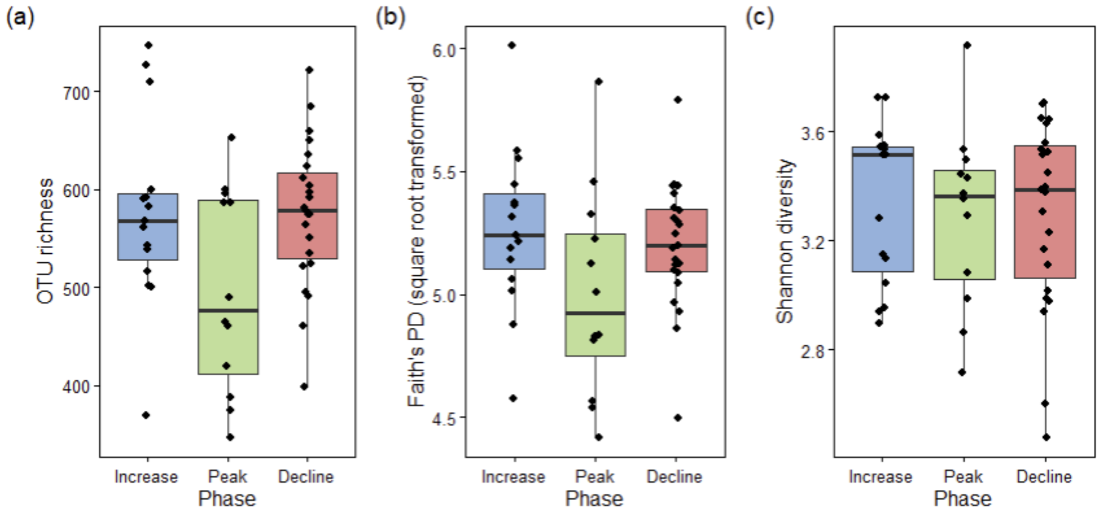

Figure S5. (a) OTU richness, (b) Faith's phylogenetic diversity (square root transformed), and (c) Shannon diversity in the snowshoe hare fecal bacterial microbiome (50 samples) across phases of the population cycle (increase, peak, decline). Bold horizonal lines represent medians, boxes represent the interquartile range (IQR), whiskers represent the Q1 or Q3 ± 1.5 x IQR, and points represent individual samples.

MOLECULAR ECOLOGY

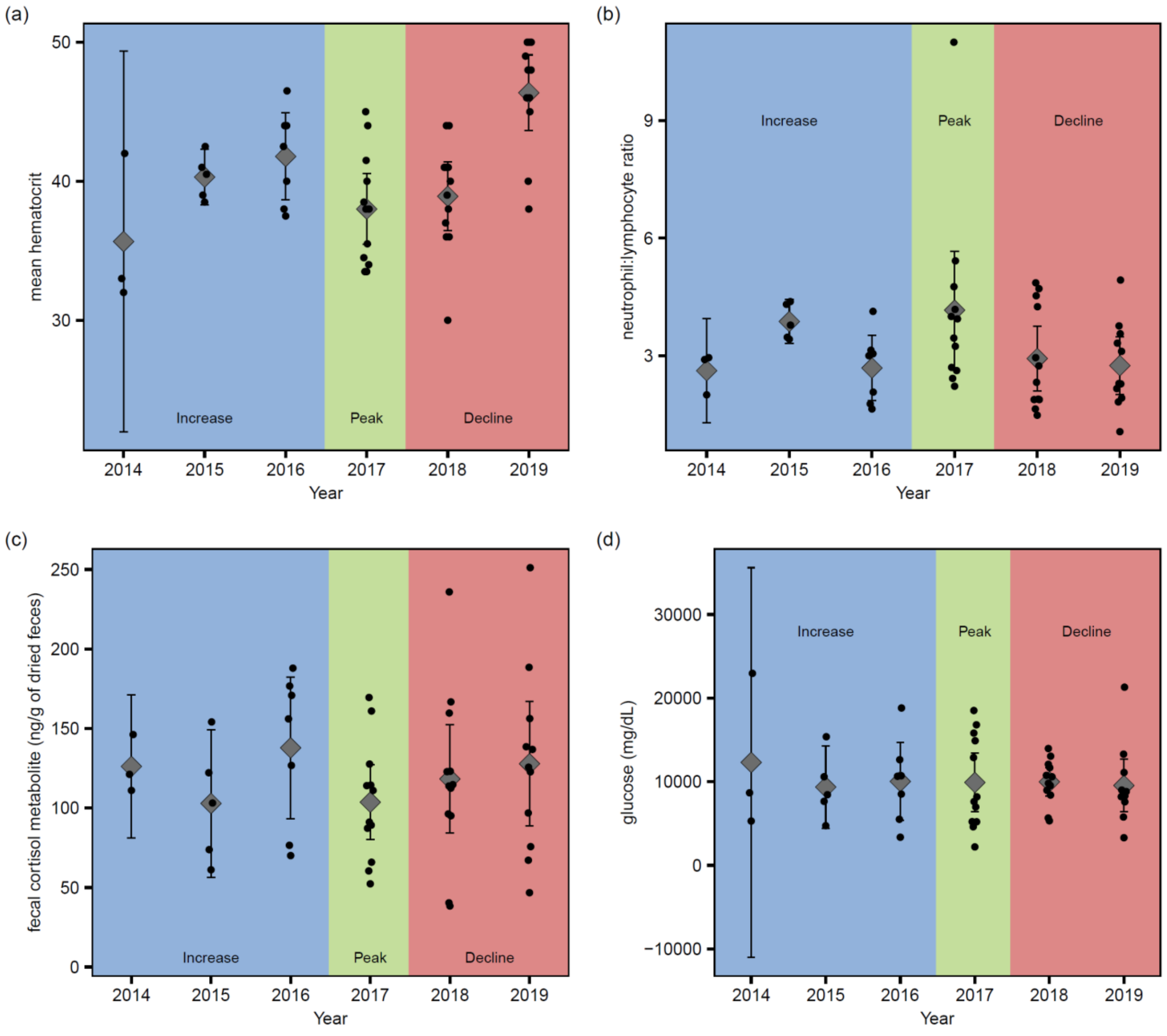

Figure S6. Plots of mean (a) blood hematocrit, (b) neutrophil:lymphocyte ratio, (c) fecal cortisol metabolite concentration, and (d) blood glucose concentration from ACTH-Dexamethasone hormone challenges (± 95% confidence intervals) collected during each February from 2014–2019. Circular points represent individual samples. Panel colours represent population cycle phase (blue denotes the increase; green the peak; red the decline).

MOLECULAR ECOLOGY

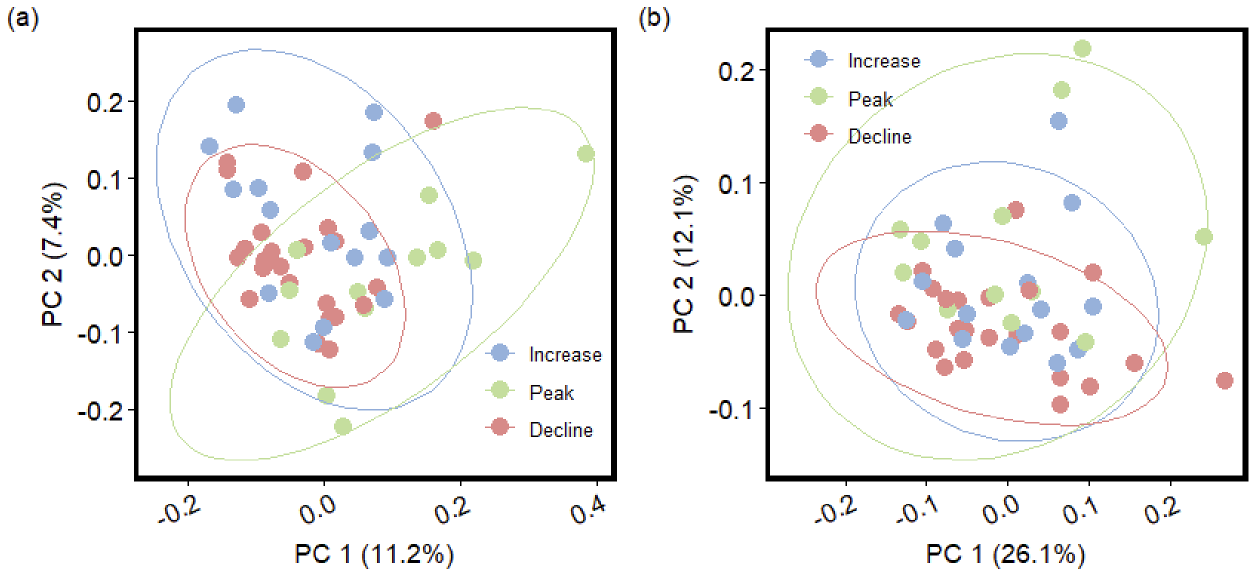

Figure S7. Principal coordinate analysis ordinations of (a) UniFrac and (b) weighted UniFrac distances in the snowshoe hare fecal bacterial microbiome (OTU-level) across phases of the hare population cycle (50 samples). Points represent individual samples and circles denote 95% confidence ellipses.

MOLECULAR ECOLOGY

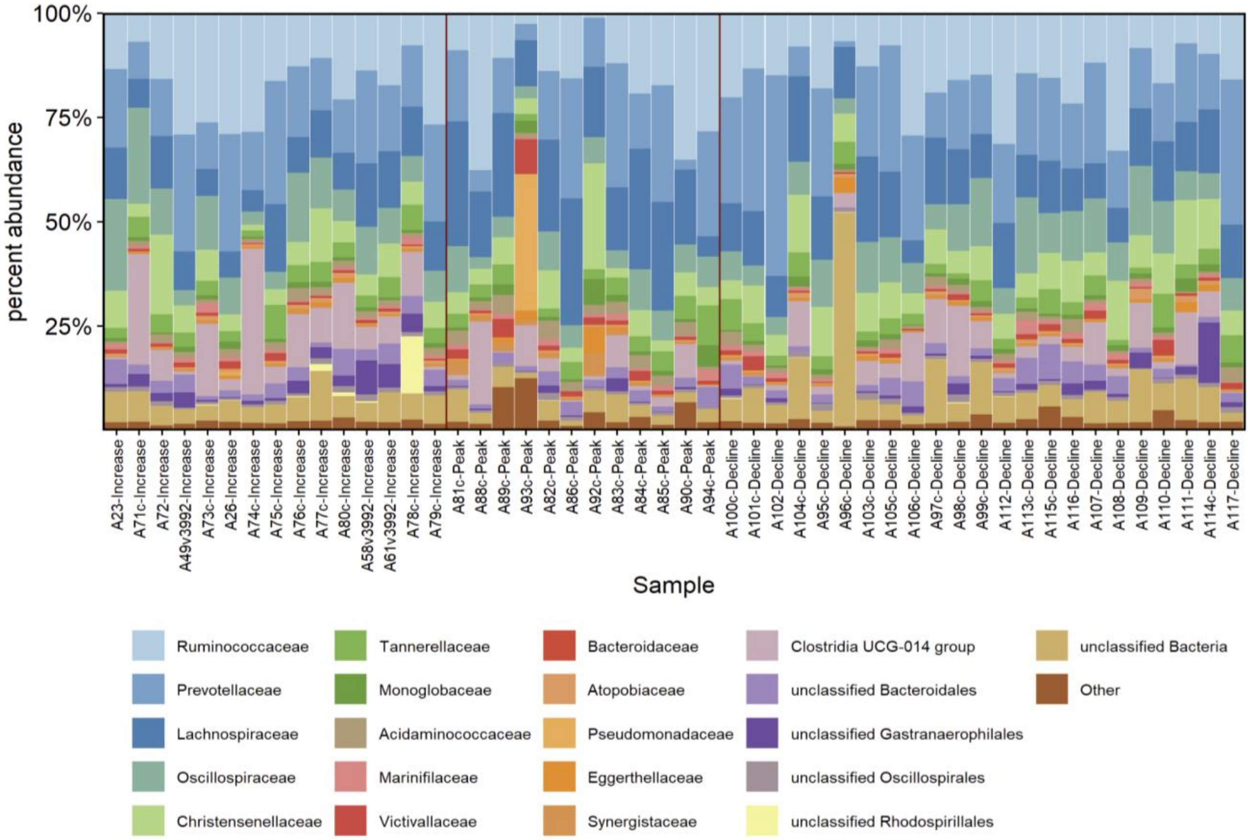

Figure S8. A barplot of bacterial taxa (family level or or coarser) in the snowshoe hare fecal microbiome. Each column represents a different individual (n = 50). Vertical red lines separate phases of the population cycle.

MOLECULAR ECOLOGY

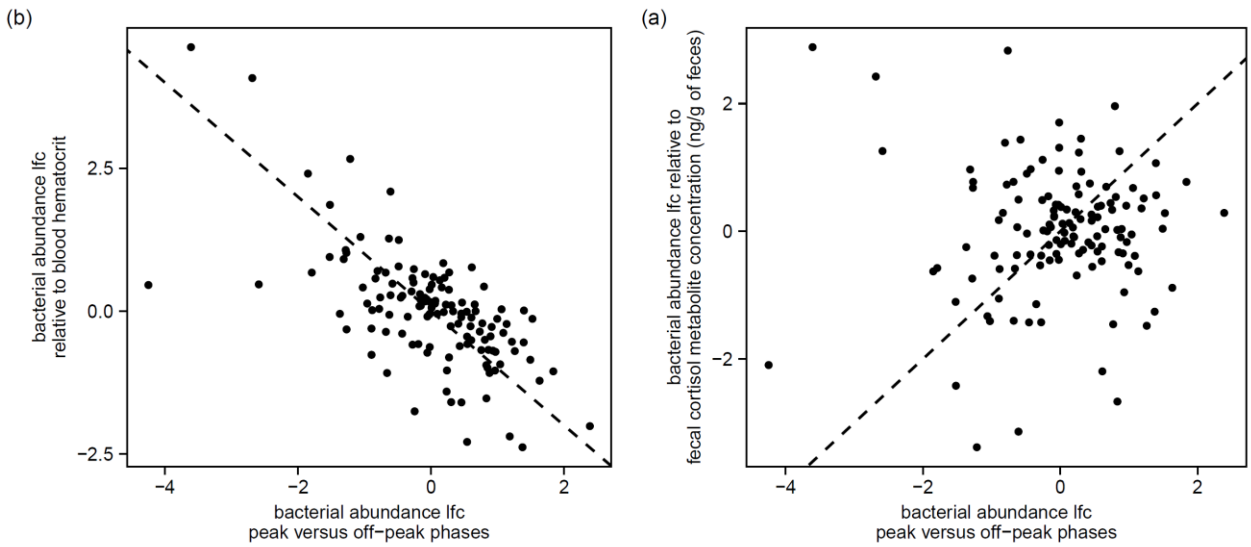

Figure S9. Estimates of log-fold change (lfc; centered and variance standardized) in bacterial taxon (family or coarser) abundance in the snowshoe hare fecal microbiome (n = 50) between peak and off-peak phases of the population cycle, versus the relationships of those same bacterial taxa with (a) blood hematocrit and (b) fecal cortisol metabolite concentration. Estimates obtained from analysis of compositions of microbiomes tests. Points represent bacterial families, or coarser taxonomic groupings representing the finest taxonomic level to which OTUs could be assigned. Dotted lines denote the 1:1 (or inverse 1:1) lines.

MOLECULAR ECOLOGY

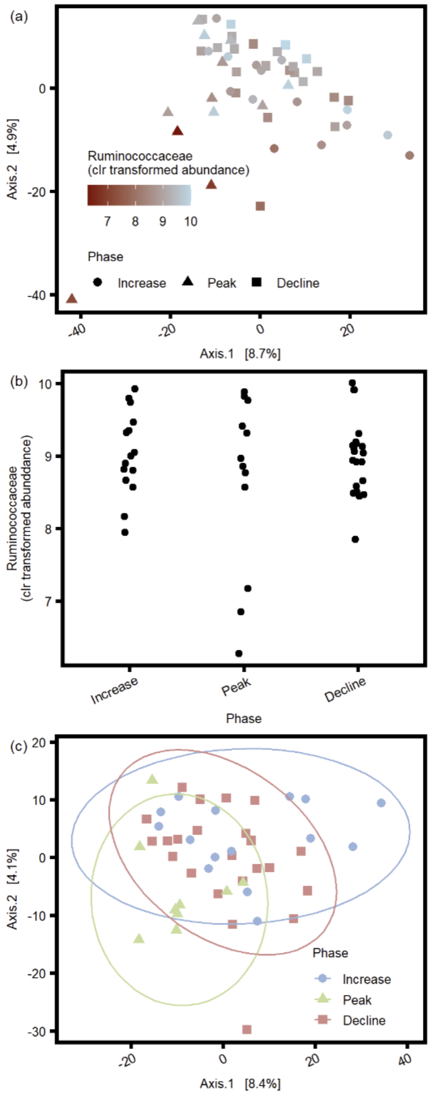

Figure S10. (a) a principal coordinate ordination of OTU level Aitchison distance in the snowshoe hare fecal bacterial microbiome (n = 50) coloured by centered log ratio (clr) transformed Rumunicoccaceae abundance. (b) a jitter plot of clr transformed Ruminococcaceae abundance in the snowshoe hare fecal bacterial microbiome (n = 50) across phases of the hare population cycle. (c) a principal coordinate ordination of OTU level Aitchison distance in the snowshoe hare fecal bacterial microbiome, with low Ruminococcaceae abundance outliers removed (n = 47). Ellipses denote 95% confidence intervals. Despite removal of outliers, Aitchison distances differed between peak and increase (R^2 = 0.07, $F_{2,32}$ = 1.703, p = 0.002), and peak and decline phases (R^2 = 0.05, $F_{2,32}$ = 1.496, p = 0.0005), but not between increase and decline phases (R^2 = 0.03, $F_{2,32}$ = 1.184, p = 0.06), among pairwise adonis comparisons.

MOLECULAR ECOLOGY

Supporting Information References

O'Donoghue, M., Boutin, S., Krebs, C.J., & Hofer, E.J. (1997). Numerical responses of coyotes and lynx to the snowshoe hare cycle. *Oikos*, **80**, 150–162. https://doi.org/10.2307/3546526
